# Angiotensin II-Induced Vasoconstriction via Rho Kinase Activation in Pressure-Overloaded Rat Thoracic Aortas

**DOI:** 10.3390/biom11081076

**Published:** 2021-07-21

**Authors:** Yuka Terada, Katsutoshi Yayama

**Affiliations:** Laboratory of Cardiovascular Pharmacology, Department of Biopharmaceutical Sciences, Kobe Gakuin University, Minatojima 1-1-3, Chuo-ku, Kobe 650-8586, Japan; y.terada@pharm.kobegakuin.ac.jp

**Keywords:** angiotensin II, abdominal aortic banding, Rho kinase, Janus kinase, myosin light chain phosphatase

## Abstract

Angiotensin II (Ang II) induces vasoconstriction through myosin light chain (MLC) kinase activation and MLC phosphatase inactivation via phosphorylation of myosin phosphatase targeting subunit 1 (MYPT1) by Rho kinase. However, the detailed mechanism underlying Rho kinase activation by Ang II is still unknown. We investigated the mechanism of Ang II-induced vasoconstriction mediated by Rho kinase in pressure-overloaded rat thoracic aortas. Pressure-overloaded rats were produced by coarctation of the suprarenal abdominal aorta in four-week-old male Wistar rats. The contractile response to Ang II was significantly enhanced in the pressure-overloaded rats. Ang II-induced vasoconstriction was attenuated by inhibitors of Rho kinase, extracellular signal-regulated kinase 1 and 2 (Erk1/2), and epidermal growth factor receptor (EGFR) in both the sham-operated and pressure-overloaded rats. The Ang II-induced vasoconstriction was attenuated by a Janus kinase 2 (JAK2) inhibitor in only the pressure-overloaded rats. The protein levels of MYPT1 and JAK2 increased only in the pressure-overloaded rat thoracic aortas. These results suggested that Ang II-induced contraction is mediated by Rho kinase activation via EGFR, Erk1/2, and JAK2 in pressure-overloaded rat thoracic aortas. Moreover, Ang II-induced contraction was enhanced in pressure-overloaded rats probably because the protein levels of MYPT1 and JAK2 increased in the thoracic aortas.

## 1. Introduction

Hypertension is associated with endothelial dysfunction and enhanced vascular reactivity to vasoconstrictor stimuli. Pro-hypertensive stimuli, such as activation of the renin-angiotensin system, stimulate vascular smooth muscle cell (VSMC) signaling, which promotes vasoconstriction, vascular hypertrophy, fibrosis, inflammation, and calcification, processes that underlie vascular functional, structural, and mechanical changes in hypertension [1].

Angiotensin II (Ang II) is a key factor in the development of hypertension and induces vasoconstriction via type-1 (AT_1_) receptor which involves the increase in intracellular Ca^2+^ concentration. Increase in intracellular Ca^2+^ concentration forms a Ca^2+^/calmodulin complex, which activates myosin light chain (MLC) kinase (MLCK), causes MLC phosphorylation, and subsequent smooth muscle contraction. On the other hand, Ang II activates Rho kinase and causes MLC phosphatase (MLCP) inactivation through phosphorylation of myosin phosphatase targeting subunit 1 (MYPT1) and leading to the Ca^2+^ sensitization and vasoconstriction [2,3]. Rho kinase is activated by RhoA, a member of the Rho family of small GTPase-binding protein, that is activated by guanine nucleotide exchange factors (GEFs). However, the detailed mechanism of Rho kinase activation by Ang II is still unknown.

Furthermore, many studies revealed that Ang II stimulates phosphorylation of many non-receptor tyrosine kinases and influences activity of receptor tyrosine kinases, leading to vasoconstriction [4,5]. Ang II activates Src, epidermal growth factor receptor (EGFR), and extracellular signal-regulated kinase 1 and 2 (Erk1/2), and induces VSMC proliferation and vasoconstriction [6,7,8,9,10,11]. In addition, Ang II transactivates EGFR via phosphorylation by Src at the cytoplasm site [6,12,13] or the metalloproteinase-dependent shedding of the heparin-binding EGF-like growth factor (HB-EGF) [9,14]. Moreover, it has been reported that Janus kinase 2 (JAK2) is involved in Ang II-induced Rho kinase activation and contraction via activation of Arhgef1 [15].

We reported that plasma renin concentration and activity increased in pressure-overloaded rats by abdominal aortic banding [16]. Additionally, Ang II-evoked contraction decreased in pressure-overloaded rat thoracic aortas at 7 days after banding operation, because the type-2 (AT_2_) receptor mRNA increased in pressure-overloaded thoracic aortas [16]. However, it is not clear whether Ang II-evoked contraction in pressure-overloaded rat thoracic aortas at later timepoints after the banding operation is also decreased or not. Moreover, the mechanisms of Ang II-evoked contraction and Rho kinase activation in pressure-overloaded thoracic aortas are unknown.

In the present study, we compared the different contractile responses to Ang II in the thoracic aortas of both sham-operated and pressure-overloaded rats at 28 days after banding operation. In addition, we investigated whether Ang II-induced contraction in pressure-overloaded rat thoracic aortas involves Src, EGFR, metalloproteinase, Erk1/2, JAK2, or Rho kinase, and whether expression levels of Src, Erk1/2, JAK2, and MYPT1 in thoracic aortas are different between sham-operated and pressure-overloaded rats.

## 2. Materials and Methods

### 2.1. Chemicals

Ang II was purchased from Peptide Institute, Inc. (Osaka, Japan). All inhibitors were purchased from Merck-Millipore (Tokyo, Japan). The inhibitors (product name, chemical name and concentration used) are described in Table 1. The concentration of inhibitors was selected based on our previous studies [17,18].

### 2.2. Animals and Abdominal Aortic Banding Operation

All animal experiments were performed according to the guidelines of the Kobe Gakuin University Experimental Animal Care and Use Committee (16-03, 11 July 2016, A17-14, 6 April 2017, A18-12, 1 April 2018 and A19-15, 1 April 2019). Four-week-old male Wistar rats (Japan SLC, Hamamatsu, Japan) were subjected to abdominal aortic banding or sham operation, as described in the previous study [16]. We understand that males are commonly used to avoid the effects of sex hormones. Briefly, the aorta was exposed through a midline abdominal incision. Then, a blunt 22-gauge needle was placed adjacent to the abdominal aorta between the renal arteries just below the renal bifurcations. A ligature was tightened around the aorta and adjacent needle, and then the needle was carefully pulled out. In the sham operation groups, only a midline incision of approximately the same size was made, but the aorta was not ligated.

### 2.3. Organ Chamber Experiments

After 4 weeks of abdominal aortic-banding operations, the rats were euthanized by bleeding from the carotid arteries under isoflurane anesthesia. The thoracic aortas were excised and immediately placed in Krebs-Henseleit solution of the following composition (mmol/L): NaCl 118.4, KCl 4.7, CaCl_2_ 2.5, KH_2_PO4 1.2, MgSO_4_ 1.2, NaHCO_3_ 25 and glucose 11.1. The aortas were cleaned of adherent connective tissue and cut into rings (3 mm long). Each ring was fixed vertically under a resting tension of 1 g in a 5-mL organ chamber filled with the Krebs-Henseleit solution (37 °C, pH 7.4), as described previously [19]. The chamber solution was continuously aerated with a gas mixture of 95% O_2_/5% CO_2_ and the rings were allowed to equilibrate for 1 h before the start of the experiments. The isometric tension was measured as described previously [17,18,19]. After equilibration, Ang II was cumulatively added to the chamber solution at a final concentration of between 0.1 nmol/L and 1 μmol/L. The inhibitors were dissolved in dimethyl sulfoxide and 10 µL of solution was added to the chamber 15 min prior to Ang II. The Magnus measure we used in the experiment consisted of eight chambers. Chamber A was for control (cumulative addition of Ang II), and chambers B to F were pretreated with an inhibitor, and then Ang II was cumulatively added. The figure was created based on the contraction data obtained from each chamber. The contractile responses observed were expressed as a percentage of the maximal contraction evoked by 40 mmol/L KCl. KCl was added before treatment with inhibitors.

### 2.4. Western Blotting

4 weeks after the abdominal aortic-banding operation, the thoracic aortas were isolated and homogenized in 700 μL lysis buffer consisting of 50 mmol/L Tris-HCl (pH 7.4), 150 mmol/L sodium chloride, protease inhibitor cocktail (Nacalai Tesque, Kyoto, Japan), and phosphatase inhibitor cocktail (Nacalai Tesque, Kyoto, Japan). Samples were centrifuged at 12,000× *g* for 10 min at 4 °C, and the concentration of soluble protein in the supernatant was measured by bicinchoninic acid protein assay (Thermo Scientific, Waltham, MA, USA). Then, 10 μg of protein was loaded in each well and separated by sodium dodecyl sulfate-polyacrylamide gel electrophoresis, followed by transfer to polyvinylidene fluoride membranes (Immobilon-P^®^; Millipore, Billerica, MA, USA). The blots were blocked with 1% bovine serum albumin in Tris-buffered saline (10 mmol/L Tris in 100 mmol/L NaCl containing 0.1% Tween 20, pH 7.5). The membrane was cut into two and incubated with different antibodies. To specifically detect total or phosphorylated protein, blots were incubated with primary antibodies available commercially. The primary antibodies used in this study are detailed in Table 2. Then the bound antibodies were detected by peroxidase-conjugated anti-rabbit or mouse IgG antibodies in the Chemi-Lumi One Super system (Chemi-Lumi One Super; Nacalai Tesque, Kyoto, Japan). Immunoblots were quantified using densitometry with ChemiDoc Touch MP (Bio-Rad Laboratories, Hercules, CA, USA) and Image Lab software (Bio-Rad Laboratories, Hercules, CA, USA). The original data is shown as Supplementary Materials (Figures S1–S4).

### 2.5. Statistical Analysis

All data are expressed as mean ± standard error of the mean (S.E.M.). Statistical comparisons were performed using Mann-Whitney U test with pairwise comparisons. Concentration-response curves were compared using repeated-measures analysis of variance followed by Bonferroni-Dunn test using the Graph Pad Prism 6 software. Differences were considered significant at *p* < 0.05.

## 3. Results

### 3.1. Ang II-Induced Contraction in the Thoracic Aorta of Sham-Operated and Pressure-Overloaded Rats

**Figure 1:**
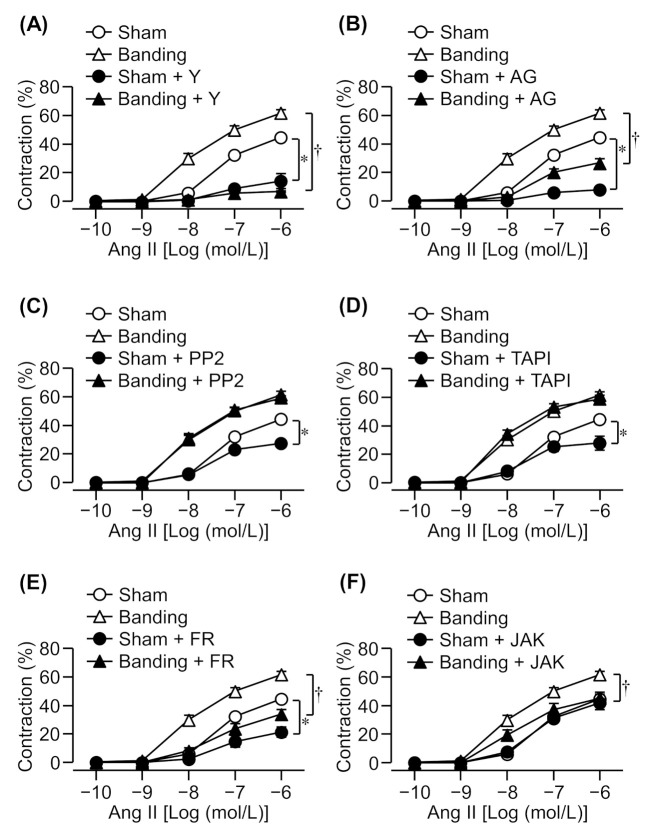
Effects of various inhibitors on angiotensin II (Ang II)-induced contraction in sham-operated (Sham) or pressure-overloaded (Banding) rat thoracic aortic rings. (**A**): Y-27632 (Y: 10 µmol/L); (**B**): AG1478 (AG: 10 µmol/L); (**C**): PP2 (3 µmol/L); (**D**): TAPI-0 (TAPI: 10 µmol/L); (**E**): FR180204 (FR: 10 µmol/L); and (**F**): JAK2 inhibitor II (JAK: 10 µmol/L) was added 15 min before Ang II treatment. The Magnus measure we used in the experiment consists of eight chambers. The thoracic aorta was removed from the rat, cut into about 10 segments, and each segment was attached to the chamber. Chamber A is for control (cumulative addition of Ang II), and chambers B to F are pretreated with an inhibitor, and then Ang II is cumulatively added. The figure was created based on the contraction data obtained from each chamber. Cumulative contraction response curves normalized to 40 mmol/L KCl-induced contraction. Data are means ± S.E.M. for thoracic aortic rings from 4−12 rats, * *p* < 0.05 vs. Sham, ^†^ *p* < 0.05 vs. Banding.

Cumulative application of Ang II induced a concentration-dependent contraction in the thoracic aortic rings of both sham-operated and pressure-overloaded rats. The Ang II-induced contraction increased to a greater extent in the pressure-overloaded group than in the sham-operated group (Figure 1).

### 3.2. Effect of a Rho Kinase Inhibitor on Ang II-Induced Contraction

It has been reported that Ang II-induced contraction in intact rats’ mesenteric arteries was mediated by Rho kinase [20], which inactivates MLCP by phosphorylation of MYPT1 [2,3]. Therefore, we evaluated the effect of a Rho kinase inhibitor, Y-27632 (10 μmol/L), on Ang II-induced contraction in pressure-overloaded thoracic aortic rings. Ang II-induced contraction was markedly reduced by a Rho kinase inhibitor in both sham-operated and pressure-overloaded rats (Figure 1A). Moreover, Ang II-induced contraction in pressure-overloaded rats was suppressed by Y-27632 to the same extent as in sham-operated rats despite being slightly enhanced in this group (Figure 1A).

### 3.3. Effect of EGFR, Src, or Metalloproteinase Inhibitors on Ang II-Induced Contraction

EGFR is transactivated via phosphorylation by Src at Tyr-845 in the cytoplasm [6,12,13] or the shedding of HB-EGF by metalloproteinase such as Adam17 [9,14]. Ang II-induced proliferation, migration, and hypertrophy of VSMCs involves EGFR transactivation by Src and the shedding of pro-HB-EGF by metalloproteinase [6,9,14]. Therefore, we examined the effect of EGFR (AG1478; 10 μmol/L), Src (PP2; 3 μmol/L), or metalloproteinase (TAPI-0; 10 μmol/L) inhibitors. Ang II-induced contraction was reduced by an EGFR inhibitor in both sham-operated and pressure-overloaded rats (Figure 1B), whereas Src and metalloproteinase inhibitors suppressed it in sham-operated rats only (Figure 1C,D).

### 3.4. Effect of an Erk1/2 Inhibitor on Ang II-Induced Contraction

There are reports which state that Ang II-induced contraction and proliferation are mediated by Erk1/2 [6,9,10,11]. Therefore, we examined whether an Erk1/2 inhibitor also reduces Ang II-induced contraction in pressure-overloaded rats. Ang II-induced contraction was significantly reduced by an Erk1/2 inhibitor, FR180204 (10 μmol/L), in both sham-operated and pressure-overloaded rats (Figure 1E).

### 3.5. Effect of a JAK2 Inhibitor on Ang II-Induced Contraction

It has been reported that JAK2 activates Rho kinase through Arhgef1 and RhoA in Ang II-induced contraction [15]. Therefore, we evaluated the effect of a JAK2 inhibitor, JAK2 inhibitor II (10 μmol/L), on Ang II-induced contraction. Ang II-induced contraction was significantly reduced by a JAK2 inhibitor in pressure-overloaded rats, but not in sham-operated rats (Figure 1F).

### 3.6. Expression and Phosphorylation of MYPT1, Src, Erk1/2, and JAK2 in Thoracic Aorta

**Figure 2:**
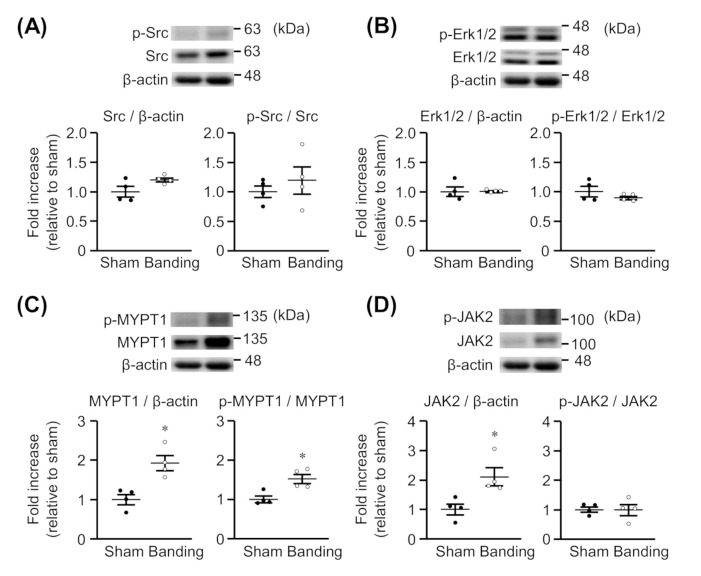
Expression of total and phosphorylated Src (**A**), Erk1/2 (**B**), MYPT1 (**C**), and JAK2 (**D**) in thoracic aortas from sham-operated (Sham) or pressure-overloaded (Banding) rats. Upper: representative blots. Lower: scatter plots demonstrating densitometric quantification of the ratios of total or phosphorylated proteins to the corresponding β-actin or total proteins, respectively. Results are shown as the fold increase relative to Sham. Data are means ± S.E.M. of four independent experiments, * *p* < 0.05 vs. Sham.

We assessed whether the expression and phosphorylation of Src, Erk1/2, MYPT1, and JAK2 would be changed in thoracic aortas by pressure overload. As shown in Figure 2A,B, the protein expression and phosphorylation of Src and Erk1/2 were not significantly different between sham-operated and pressure-overloaded rats. On the other hand, the protein expression of MYPT1 (Figure 2C) and JAK2 (Figure 2D) was significantly greater in pressure-overloaded rats than in sham-operated rats. Moreover, MYPT1 phosphorylation was enhanced in pressure-overloaded rats (Figure 2C), but the JAK2 phosphorylation was not (Figure 2D). The raw data are indicated in Supplementary Materials (Figures S1–S4).

## 4. Discussion

**Figure 3:**
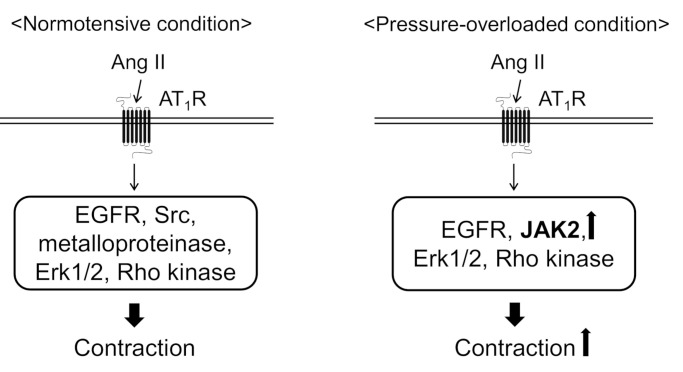
Proposed mechanisms underlying angiotensin II (Ang II)-induced aortic contraction in normotensive or pressure-overloaded condition. In normotensive condition, Ang II induces aortic contraction via epidermal growth factor receptor (EGFR), Src, metalloproteinase, extracellular signal-regulated kinase 1 and 2 (Erk1/2), and Rho kinase. In pressure-overloaded condition, Ang II induces aortic contraction via EGFR, Erk1/2, Rho kinase, and Janus kinase 2 (JAK2). Moreover, Ang II-induced aortic contraction is augmented in pressure-overloaded condition probably because the protein expression of JAK2 and myosin phosphatase targeting subunit 1 is increased. AT_1_R, Ang II type 1 receptor.

In the present study, we found that abdominal aortic banding increased the contractile response to Ang II in thoracic aortas. Because the protein expression of JAK2 and MYPT1 increased in the thoracic aortas from pressure-overloaded rats, it seems likely that Ang II-induced contractile signaling is facilitated by JAK2 and excess MLCP inactivation mediated through MYPT1 phosphorylation by Rho kinase. We also revealed that Ang II-induced contraction was mediated by EGFR, Erk1/2, and Rho kinase in the both the sham-operated and pressure-overloaded thoracic aortic rings (Figure 3). On the other hand, Src and EGFR transactivation, via HB-EGF shedding by metalloproteinase, were involved in only sham-operated rat thoracic aortas (Figure 3).

Many studies revealed that increased Ca^2+^ signaling, vascular hyperreactivity, and an exaggerated contractile response to vasoactive agents are demonstrated in hypertensive models. Vascular contraction occurs as a result of both Ca^2+^ release from the sarcoplasmic reticulum via inositol triphosphate receptors and ryanodine receptors, and extracellular Ca^2+^ influx through voltage-dependent, receptor-operated, transient receptor potential and store-operated channels. In the mesenteric arteries taken from spontaneously hypertensive rats (SHR), currents carried by L-type Ca^2+^ channels were larger and their voltage-dependence shifted in the negative direction compared to Wistar-Kyoto rats (WKY) [21]. The contractile tension in response to extracellular Ca^2+^ in the endothelium-denuded mesenteric arteries was greater in Ang II-induced hypertensive rats compared with sham-operated normotensive rats [22]. This evidence is consistent with our results that Ang II-induced contraction in pressure-overloaded rat thoracic aortas increased as compared with sham-operated rats. We previously reported that Ang II-induced contraction was reduced in the thoracic aortas from pressure-overloaded rats at 7 days after the abdominal aortic-banding operation because of the increase in AT_2_ receptor mRNA levels, which negatively modulated the vasoconstrictor sensitivity [16]. The increase in AT_2_ receptor mRNA levels persisted for 28 days after the banding operation [16]. We considered that the contractile signaling via the AT_1_ receptor was facilitated by long-lasting pressure overload, possibly not being suppressed by AT_2_ receptor signaling, and that Ang II-induced contraction increased. Although the activation of Rho kinase was found to be involved in the AT_1_ receptor-mediated contraction by Ang II in pressure-overloaded aortas, it was not possible to clarify whether the AT_2_ receptor modified this system. This issue must be clarified in the next study.

Ca^2+^ sensitization by RhoA/Rho kinase plays an important role in vasoconstriction. RhoA is activated by GEFs such as Arhgef1, which are activated by the binding of the activated α subunit of G_12/13_ and phosphorylation by several tyrosine kinases, such as proline-rich tyrosine kinase 2 [23]. There is evidence that Ang II activates Arhgef1 and induces contraction via Rho kinase in animals [15,20]. Ang II also activates Arhgef1 in human coronary artery smooth muscle cells [24]. Furthermore, GEFs/RhoA/Rho kinase pathway is enhanced in experimental hypertensive models and hypertensive patients. The protein expression of the RhoA active form, RhoA membrane translocation implying RhoA activation, and phosphorylation of MYPT1 increased in the aortas or VSMC from hypertensive models [25,26,27], and p63RhoGEF mRNA and protein expression increased in the peripheral blood mononuclear cells from essential hypertensive patients [28]. We revealed that Ang II-induced contraction was mediated by Rho kinase in both the sham-operated and pressure-overloaded rats. We also found that Ang II-evoked contraction in pressure-overloaded rats was almost completely suppressed by a Rho kinase inhibitor, Y-27632. Moreover, we found that the protein expression and phosphorylation of MYPT1 increased in pressure-overloaded aortas. These results suggest that Rho kinase increased MYPT1 phosphorylation, that is, it inactivates MLCP and strongly contributes to Ang II-induced contraction in pressure-overloaded vessels.

EGFR was transactivated via phosphorylation at Tyr-845 in the cytoplasm by Src [6,12,13] or the shedding of HB-EGF by metalloproteinase [9,14]. Ang II-induced proliferation, migration, and hypertrophy of VSMCs were mediated by EGFR transactivation [6,9,14]. EGFR was also involved in Ang II-induced cardiac hypertrophy and hypertension [29,30]. In addition, Ang II-stimulated Src and EGFR phosphorylation was augmented in VSMCs from SHR compared with VSMCs from WKY, which were attenuated by an EGFR inhibitor, AG1478 [31]. Moreover, Src is implicated in the Ang II-induced increase in intracellular Ca^2+^ concentration in human VSMCs and vasoconstriction in rats’ mesenteric arteries [8]. In the present study, we demonstrated that Ang II-induced contraction was reduced by an EGFR inhibitor, AG1478, in the both sham-operated and pressure-overloaded rats. On the other hand, the contractile response to Ang II was suppressed by Src (PP2) and metalloproteinase (TAPI-0) inhibitors in sham-operated rats only, suggesting EGFR transactivation via another pathway instead of Src and the shedding of HB-EGF by metalloproteinase in Ang II-induced contraction in pressure-overloaded rat thoracic aortas. However, the mechanism of EGFR transactivation by Ang II in the pressure-overloaded aortas is not clear and further studies are needed.

Erk1/2 is one of mitogen-activated protein kinases (MAPKs) and plays an important role in cell growth in many cell types. Numerous studies revealed that Erk1/2 exists downstream of the pathway of EGFR and Src [6,7,9,10,31], and contributes to Ang II-induced proliferation of VSMCs [6,9]. Additionally, Ang II-mediated Erk1/2 phosphorylation is more augmented in VSMCs from SHR than in VSMCs from WKY [11,31]. Furthermore, it has been reported that Erk1/2 is implicated in Ang II-induced contraction in rats’ aortas and VSMCs from SHR [10,11]. We found that Ang II-induced contraction was reduced by an Erk1/2 inhibitor, FR180204, and the protein expression levels of Erk1/2 were not different between sham-operated and pressure-overloaded aortas, which is consistent with another group’s report using VSMCs from SHR [7].

The present study suggests that JAK2 plays an important role in Ang II-induced contraction in pressure-overloaded rats, because there is an increase of JAK2 in pressure-overloaded rat aortas. To the best of our knowledge, this is first report showing that JAK2 is involved in Ang II-induced contraction in hypertension model. There is evidence that AG490, a JAK2 inhibitor, reduced Ang II-induced contraction in normotensive rat thoracic aortas and suppressed Ang II-induced hypertension [15]. However, we could not obtain results regarding the reduction of Ang II-induced contraction by the JAK2 inhibitor in sham-operated rat thoracic aortas. It has been reported that AG490 can inhibit a number of other kinase signaling pathways as well, and that the JAK2 inhibitor II we used is a potent and specific inhibitor of JAK2 [32]. Therefore, it is possible that JAK2 does not participate in Ang II-induced contraction in normotensive rats. However, the difference in the mechanisms by which JAK2 affects proliferation and migration of VSMCs by Ang II [33], but not in contraction, remains unknown.

We previously found that the mRNA expression of the AT_1_ receptor was not different between sham-operated and pressure-overloaded rat thoracic aortas [16], suggesting that the downstream signaling of the AT_1_ receptor is augmented by pressure overload. In this study, we suggest that pressure overload results in hyperreactivity against Ang II because of an increase in the expression of MYPT1 and JAK2. However, the mechanism by which this expression increases is unknown. In addition, the activation mechanism of EGFR, Erk1/2, JAK2, and Rho kinase, which has been shown to be involved in Ang II-induced contraction in pressure-overloaded rats, has not been clarified. We reported that OVA-induced Rho kinase activation involved Src, EGFR, and Erk1/2 [17,18]. In addition, Src phosphorylates and activates EGFR, and EGFR activation causes Erk1/2 activation in OVA-stimulated rats’ mesenteric arteries and mice aortas [17,18], which is consistent with other reports where VSMCs were stimulated with Ang II [6,7,9,10,31]. Furthermore, JAK2 has already been reported to play a role in Rho kinase activation [15]. These reports give the suggestion that EGFR, Erk1/2, and JAK2 are involved in Ang II-induced Rho kinase activation in pressure-overloaded rat thoracic aortas.

The phenylephrine- and serotonin-induced contractile responses in coronary and carotid arteries was significantly inhibited by treatment with a Rho kinase inhibitor in SHR but not those in WKY [34]. The Rho kinase inhibitor induced vasodilator response in the forearm significantly greater in hypertensive patients than in normal subjects [35]. The administration of the Rho kinase inhibitor to SHR or deoxycorticosterone acetate-salt hypertensive rats, but not normotensive rats, significantly reduced blood pressure. Taken together, it is suggested that Rho kinase could be regarded as a novel therapeutic target for the treatment of hypertension [36]. In the future, it is necessary to investigate the detailed Ang II-induced contraction mechanism through the activation of Rho kinase and JAK2 in hypertensive patients.

## 5. Conclusions

The findings of this study suggest that Ang II-mediated contraction involves EGFR, ERK1/2, JAK2, and Rho kinase in the thoracic aortas of pressure-overloaded rats. Moreover, Ang II-induced contraction is augmented in pressure-overloaded thoracic aortas and may be partly due to an increase in MYPT1 and JAK2 expression levels. Because Ang II plays a pivotal role in the vascular function, an improvement in the signaling may represent a beneficial target for the treatment of hypertension.

## Figures and Tables

**Table 1:** Inhibitors used in this study.

Product Name	Chemical Name	Concentration Used (µM)
AG1478	4-(3-chloroanilino)-6,7-dimethoxyquinazoline	10
FR180204	5-(2-phenyl-pyrazolo[1,5-a]pyridin-3-yl)-1h-pyrazolo[3,4-c]pyridazin-3-ylamine	10
JAK2 Inhibitor II	1,2,3,4,5,6-hexabromocyclohexane	10
PP2	4-amino-3-(4-chlorophenyl)-1-(t-butyl)-1h-pyrazolo[3,4-d]pyrimidine	3
TAPI-0	n-(R)-(2-[hydroxyaminocarbonyl]methyl)-4-methylpentanoyl-l-naphthylalanyl-l-alanine amide	10
Y-27632	R-(+)-trans-n-(4-pyridyl)-4-(1-aminoethyl)-cyclohexanecarboxamide	10

**Table 2:** Primary antibodies used in this study.

Antibody	Dilution	Catalog No.	Purchased from
MYPT1	1:600	#2634	Cell Signaling Technology (Danvers, MA, USA)
Thr-853-phosphorylated MYPT1	1:250	CSB-PA020015	CUSABIO (Houston, TX, USA)
Erk1/2	1:5000	#4695	Cell Signaling Technology (Danvers, MA, USA)
Thr-202/Tyr-204-phosphorylated Erk1/2	1:5000	#9101	Cell Signaling Technology (Danvers, MA, USA)
JAK2	1:500	#3230	Cell Signaling Technology (Danvers, MA, USA)
Tyr-1008-phosphorylated JAK2	1:200	#8082	Cell Signaling Technology (Danvers, MA, USA)
Src	1:750	#2102	Cell Signaling Technology (Danvers, MA, USA)
Tyr-416-phosphorylated Src	1:750	#2101	Cell Signaling Technology (Danvers, MA, USA)
β-actin	1:5000	A5441	Sigma-Aldrich (St. Louis, MO, USA)

## Supplementary Materials

The following are available online at https://www.mdpi.com/article/10.3390/biom11081076/s1. Figure S1: Original blots of Figure 2A, Figure S2: Original blots of Figure 2B, Figure S3: Original blots of Figure 2C, Figure S4: Original blots of Figure 2D.
