# Supplementary material for: Angiotensin II-Induced Vasoconstriction via Rho Kinase Activation in Pressure-Overloaded Rat Thoracic Aortas
Source: Biomolecules. 2021 Jul 21;11(8):1076. doi: 10.3390/biom11081076 (PMC8391281; doi:10.3390/biom11081076)
Supplement: Supplementary file 1 [file biomolecules-11-01076-s001.zip › biomolecules-1254722-supplementary.pdf]

Supplementary Materials

Angiotensin II-Induced Vasoconstriction via Rho Kinase Activation in Pressure-Overloaded Rat Thoracic Aortas

Yuka Terada and Katsutoshi Yayama *

Laboratory of Cardiovascular Pharmacology, Department of Biopharmaceutical Sciences, Kobe Gakuin University, Minatojima 1-1-3, Chuo-ku, Kobe 650-8586, Japan

*Correspondence: yayama@pharm.kobegakuin.ac.jp; Tel.: +81-78-974-4721

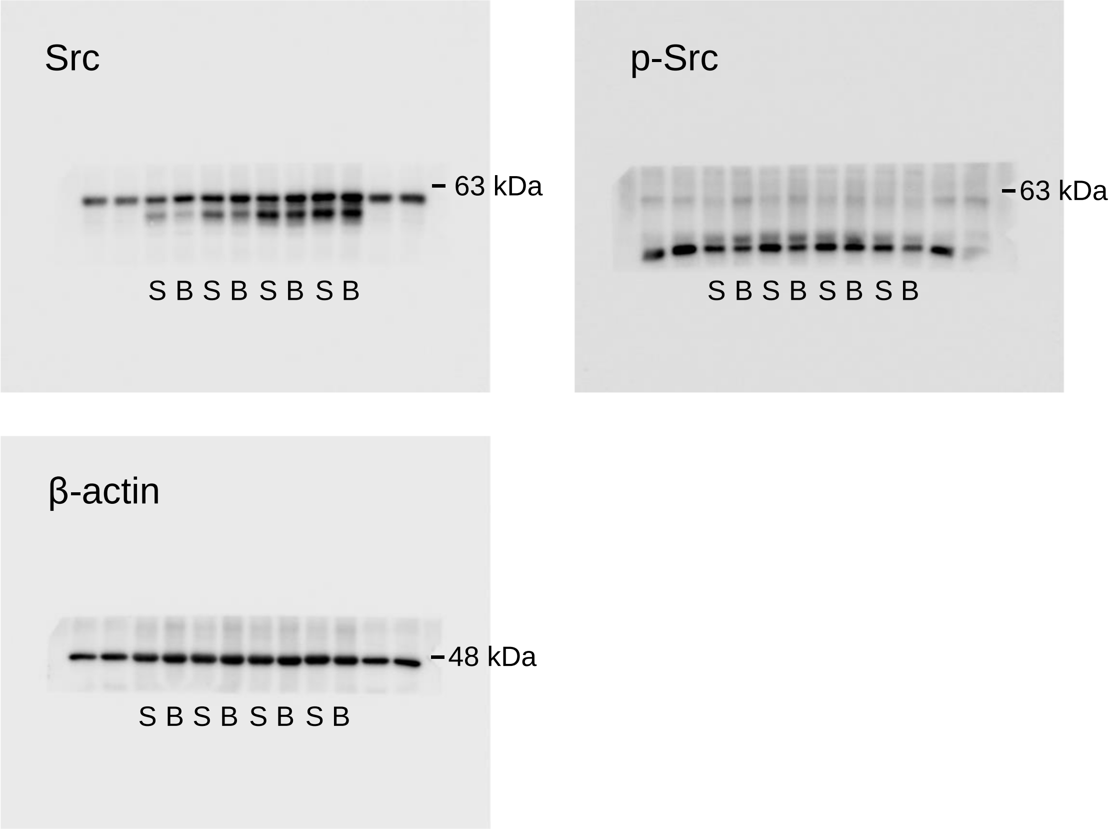

Figure S1. Original blots of Figure 2A. S: Sham, B: Banding.

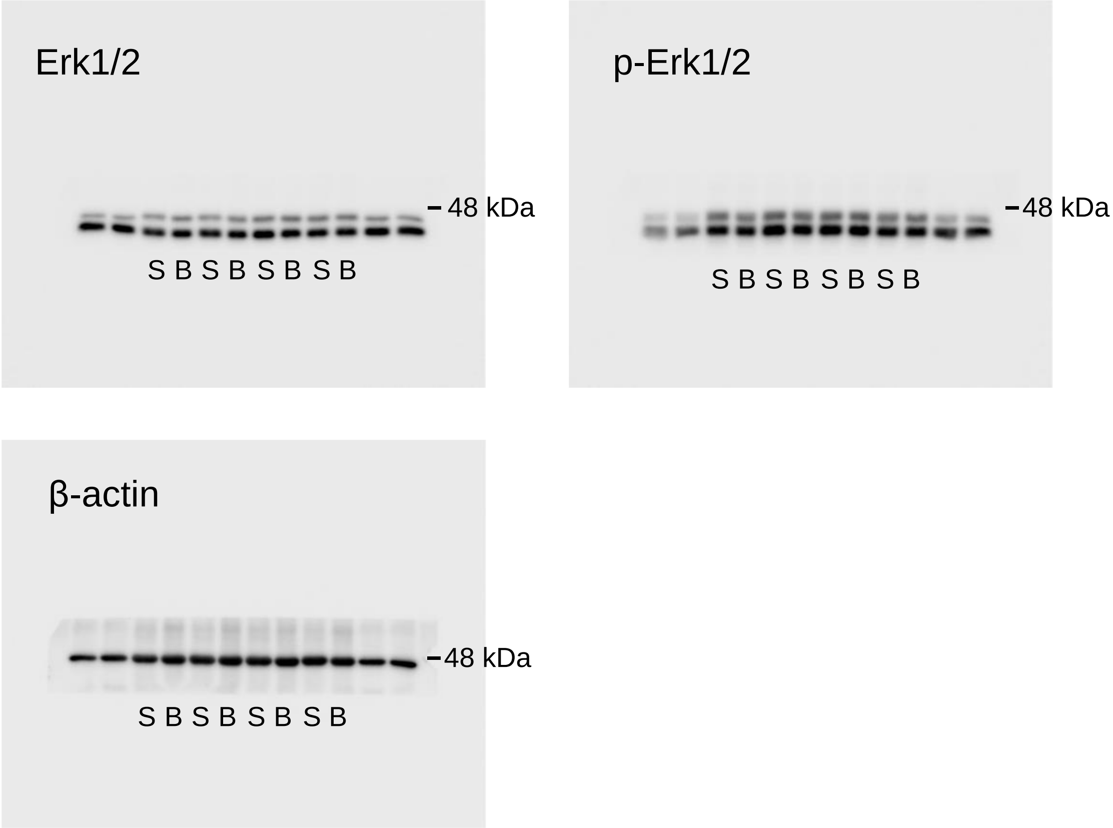

Figure S2. Original blots of Figure 2B. S: Sham, B: Banding.

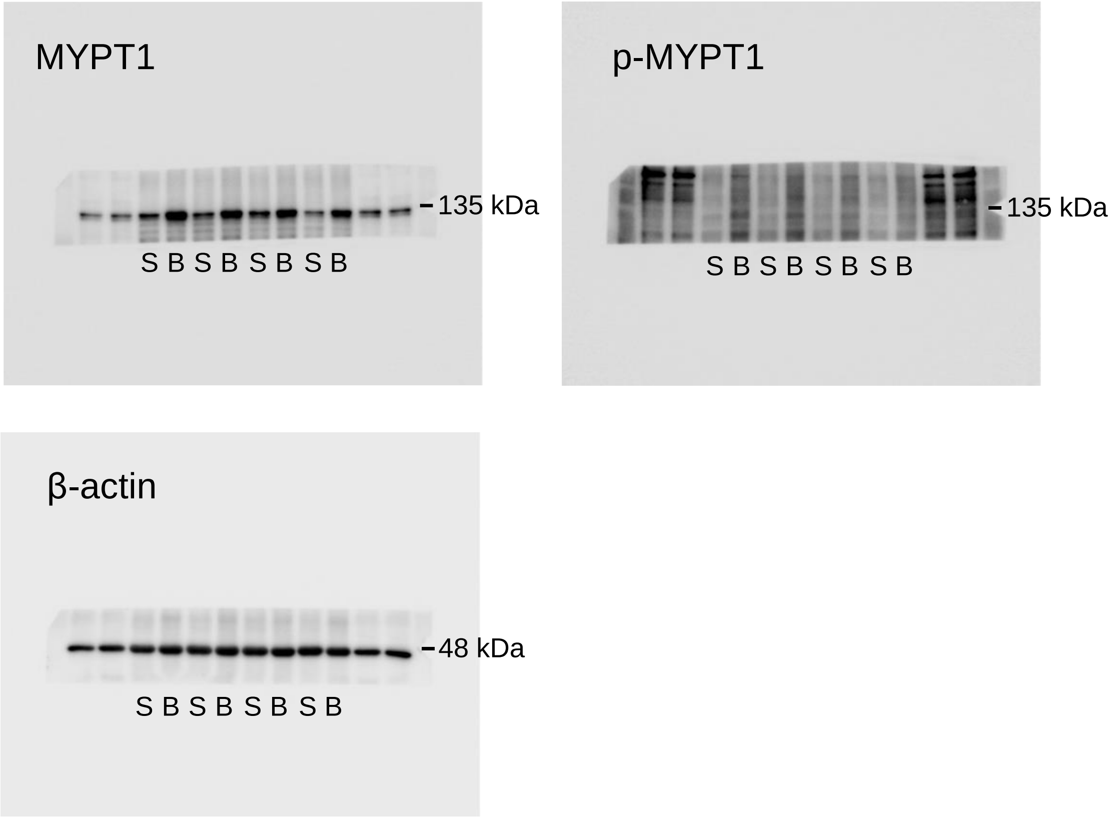

Figure S3. Original blots of Figure 2C. S: Sham, B: Banding.

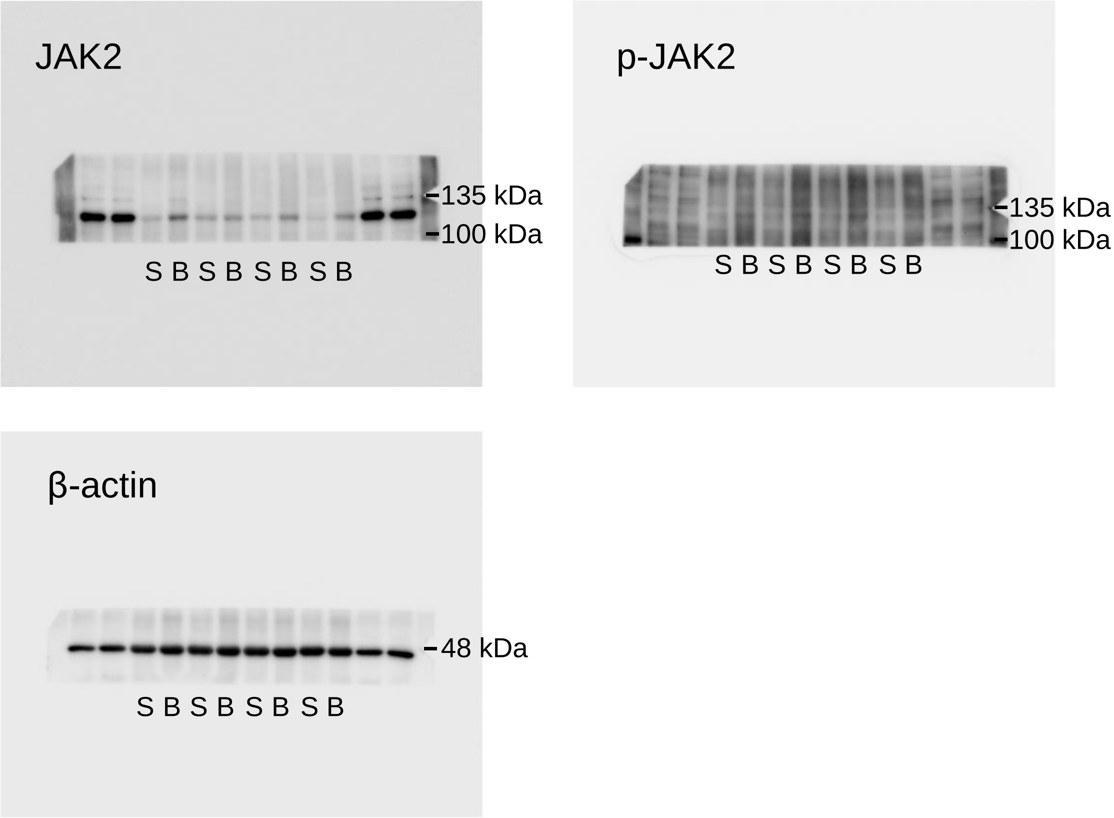

Figure S4. Original blots of Figure 2D. S: Sham, B: Banding.
